# Bilateral Pulmonary Embolism after a Short-Haul Flight in a Man with Multiple Risk Factors including Sickle Cell Trait

**DOI:** 10.1155/2017/4316928

**Published:** 2017-03-29

**Authors:** Kamille Abdool, Kanterpersad Ramcharan, Antonio J. Reyes, Nadiene Lutchman, Adrian Alexander

**Affiliations:** Department of Medicine, San Fernando Teaching Hospital, San Fernando, Trinidad and Tobago

## Abstract

We report a case of pulmonary embolism (PE) in an Afro-Caribbean man following a short commercial flight of less than 5,000 kilometers (Km) in economy class with a 1-month interval between journeys. He had an elevated body mass index (BMI) and sickle cell trait (SCT) with hyperhomocysteinemia. No other preexisting source of venous thrombosis was found. We posit that venous thromboembolism (VTE) and/or PE may have been a complication of SCT in an individual with other multiple risk factors. We discuss the possible interaction of these risk factors for VTE and/or PE and the implications for travelers at risk. The need for a PE risk score and guidelines for the prophylaxis of thromboembolism among travelers exists.

## 1. Introduction

Travel-associated PE with or without detectable deep venous thrombosis (DVT) has been documented in hospitals that are closely located to large bustling airports [1–3]. The data gathered from 3 studies at Frankfurt, Charles de Gaulle, and Madrid-Barajas airports showed a low risk of the economy class syndrome (ECS) which strictly depended on the flight distance. It was found that there was a greater risk with flight distances of greater than 5,000 km [1–3].

Multiple risk factors and preexisting comorbidities can add to the risk and prognosis. These include thrombophilia (40%), recurrent VTE (16%), Factor V Leiden (13%), VTE family history (10%), neoplastic disease (7%), and other predisposing factors such as pulmonary hypertension, Behcet's disease, use of oral contraceptives, and a BMI greater than 35 kg/m^2^ (10%) [1].

We report a case of PE in an Afro-Caribbean man following a short commercial flight of less than 5,000 km in economy class. He had an elevated BMI, SCT, and hyperhomocysteinemia as additional risk factors.

## 2. Case Presentation

A previously well, 39-year-old Afro-Caribbean man with known SCT was admitted to the hospital for worsening shortness of breath and pleuritic chest pain following a commercial flight from Trinidad to Jamaica via Miami. The travel distance of 3,528 Km was covered in a time frame of 5 hours via a break from a connecting flight. The patient had experienced shortness of breath on moderate exertion, with no chest pain or hemoptysis, during the 1-month stay in Jamaica, but he sought medical attention only in Trinidad, one month later after the return flight via the same route.

**Figure 1:**
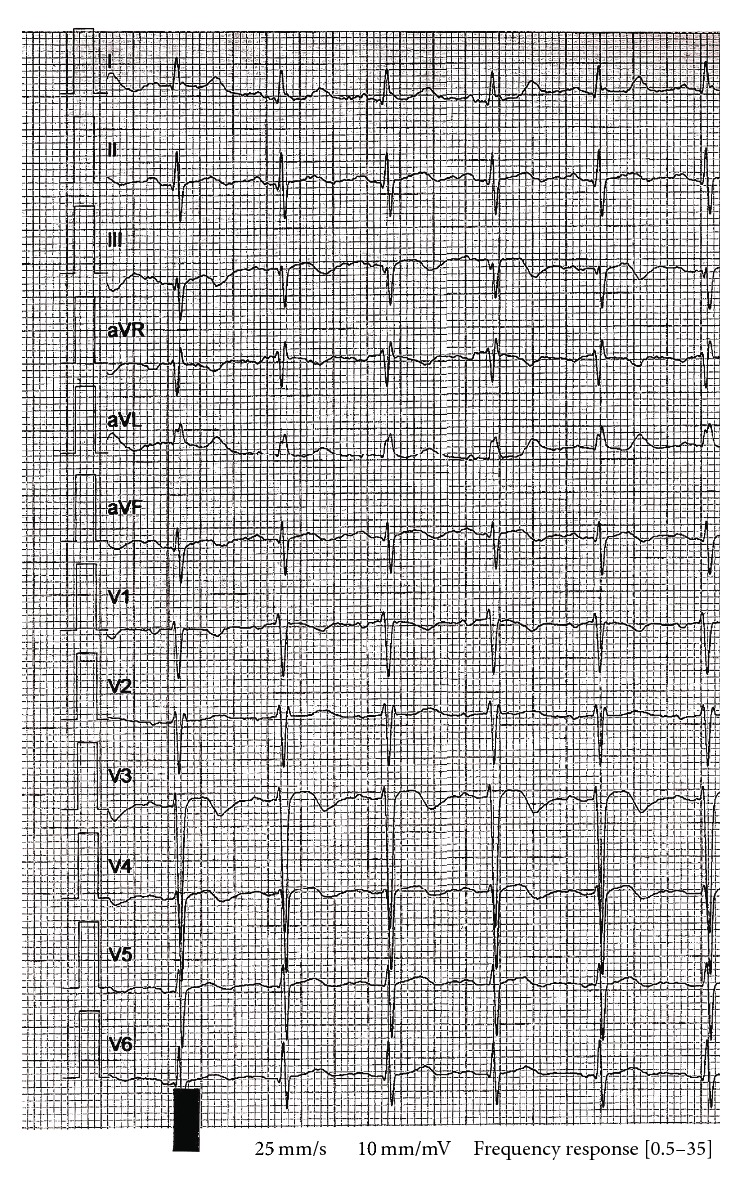
Photograph of ECG demonstrating a small Q wave with S and T waves that can be seen in lead III, the inferior leads (II, III, and aVF), T wave inversions in the right precordial leads (V1–4), and clockwise rotation-shift of the R/S transition point towards V6 with a persistent S wave in V6, all consistent with pulmonary embolism (settings: 25 mm/s and 10 mm/mV).

**Figure 2:**
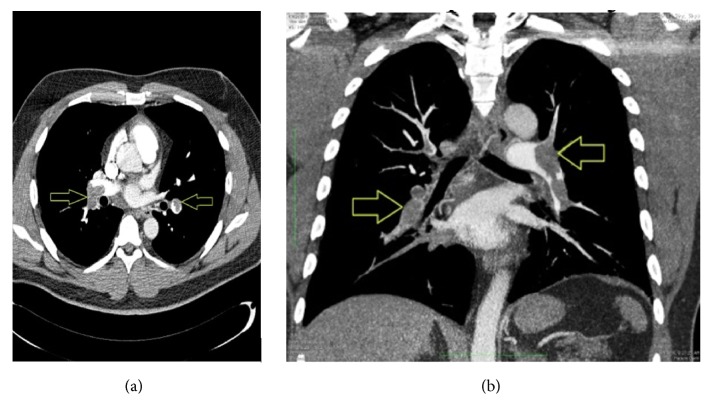
(a) CT pulmonary angiogram axial image showing a large filling defect in the right and left main pulmonary arteries affecting supply to both lobes of the lungs (see arrows). (b) CT pulmonary angiogram coronal view depicting large filling defects in the right and left main pulmonary arteries and bilateral lobar pulmonary arteries with bilateral lower lobe pulmonary atelectasis (see arrows).

There was no previous history or clinical evidence of DVT. The blood pressure was 135/79 mm Hg, the pulse was 92 beats per minute, the respiratory rate was 24 respirations per minute, and the oxygen saturation was 85% on room air which improved to 100% after increasing gradually the oxygen therapy to 15 L/min via face mask. Physical examination findings were insignificant except for a BMI of 34 kg/m^2^. Arterial blood gases on oxygen therapy showed a compensated respiratory alkalosis. The patient's Wells score was calculated as 3 which signifies a moderate risk for PE [4]. The D-Dimer test was reported to be greater than 10,000 *µ*g/L (normal < 500). An electrocardiogram (ECG) showed changes consistent with PE which reverted to normal on patient's recovery (Figure 1). Computed Tomography Pulmonary Angiogram (CTPA) showed large filling defects in both main pulmonary arteries and bilateral lobar pulmonary arteries with bilateral lower lobe pulmonary atelectasis (Figures 2(a) and 2(b)). Transthoracic echocardiography, chest X-ray, and CT scan of abdomen and pelvic were reported to be normal. Doppler ultrasound of the venous systems of both lower limbs did not show evidence of DVT. Screening for prothrombotic states was negative except for HbAS genotype (HbA: 61.2% and HbS: 36.6%) and hyperhomocysteinemia at 32 *µ*mol/L (normal < 16). Investigations for infectious, autoimmune, haematological, respiratory, cardiovascular, and mitotic diseases were negative. Serum vitamin B12 and folic acid levels were normal. Genetic testing for homocysteine polymorphisms and flow cytometry for paroxysmal nocturnal hemoglobinuria were unavailable. Diagnosed with bilateral PE, subcutaneous enoxaparin was started and, on day 2, 15 mg orally twice daily loading dose of rivaroxaban was given. On day 4, he was discharged for outpatient care. Rivaroxaban 20 mg orally daily was initiated from day 21 and continued for a year. A treadmill stress test using Bruce protocol was negative for coronary artery ischemia six weeks after discharge from hospital. One year later, the patient remains well, is off medications, and has lost 5 kilograms of body weight.

## 3. Discussion

PE or VTE has been reported after long traveling by car, bus, and train. Severe PE is extremely rare after flights of less than 8 hours [1]. VTE or PE events are more prevalent among passengers traveling long distances (>5,000 Km), with sedentary life style, poor mobility, and bended legs in economy class and/or with previous poorly controlled risk factors for thrombosis [1]. This has led to the use of the term ECS. Prophylactic measures for long-haul flights have been advocated in an attempt to minimize this risk but no risk scale has been described to guide physicians.

PE occurs frequently with no demonstrable source of primary venous thrombosis as in our patient. A meta-analysis has shown that the prevalence of DVT in suspected PE is approximately 18%, and in proven PE 36–45%. The absence of demonstrable DVT as in our case is well recognized in patients with PE. The Frankfurt study found that only 48% of patients with PE and ECS had demonstrable venous thrombosis [1].

Interestingly, the mean BMI of these patients was 35 kg/m^2^, unveiling a trend to higher values compared with ECS patients with predisposing factors (mean BMI 28; *P* = 0.364) [1]. The inclusion of the BMI as additional risk factor for PE among air travelers was emphasized because a high BMI can be a reversible risk for PE. Patients with PE due to non-air-travel ECS were significantly overweight compared with air-travel-associated PE, indicating that obesity might be a crucial risk factor for prolonged car, bus, or train traveling. Nevertheless, in patients with known risk factors for PE or VTE such as thrombophilia, positive history of VTE, or obesity, preventive measures on long-haul flights over 5,000 km should be encouraged, the authors noted [1].

Our patient had a combination of multiple risk factors for PE or VTE such as obesity, economy class travel, SCT with hyperhomocysteinemia, and African ethnicity. The patient had almost two congenital risk factors (SCT and hyperhomocysteinemia) that are per se sufficient to explain a PE. The patient had also an acquired risk factor such as two separate short-haul flights in the last four weeks. Although one cannot specify which of the congenital or acquired risk factors played a major role in this patient, the existence of multiple risk factors in a single individual traveling by air is enough evidence to support the development of PE. It is thus possible that our patient may have had subclinical calf thrombosis resulting in ECS with PE. We could not demonstrate clinical or ultrasonographic evidence of venous thrombosis as a source of embolism in this case but these observations are not unusual.

Sickle cell disease and venous thrombosis have been widely reported but the consideration of SCT as a risk factor for PE has only recently been gaining attention. SCT occurs in approximately 300 million people worldwide, with the highest prevalence of approximately 30% to 40% in sub-Saharan Africa. SCT is no longer considered a benign condition. VTE and other complications in patients with SCT have been frequently reported [5–26]. Additionally, sickle cell disease and SCT have also been associated with hyperhomocysteinemia which arguably has been associated with vascular thrombosis but is not considered a strong risk factor [10, 11]. SCT has been reported as causing venous thrombosis in many different veins in humans such as many venous beds in the legs, brain, thorax, and abdomen [12–30]. Furthermore, SCT and PE are also being linked. Massive PE has been noted previously in patients with SCT and other risk factors [28–31]. Factors contributing to such clotting tendency in HbAS patients are still unclear. Recently, the subject has been explored by studying coagulation parameters and preliminary evidence is leaning towards a prothrombotic propensity. For instance, in Nigeria, a non-“O” blood groups of individuals have been linked with thrombosis in SCT though the significance of this finding remains unknown currently [8, 32, 33].

SCT in African Americans carries a 2-fold increased risk of PE but SCT does not elevate DVT risk according to one study [6]. Neonatal screening for sickle hemoglobin conducted in the United States of America suggests that consideration should be paid to the increased PE risk of individuals with SCT [3–6, 34]. Whilst PE has been reported with air travel in HbSC disease, we have not found a previous report of PE with HbAS following air travel, suggesting low incidence and/or underdiagnosis [35, 36].

Whilst SCT may be just coincidental in our patient, there was a positive temporal relationship between a recent travel by air and PE in this patient with concurrence of other multiple risk factors. We hope by this report to increase awareness of a possible relationship between SCT and PE. So far, we are unable to say which individual risk factor was dominant in causing the ECS in our patient but in an individual with multiple risk factors for PE or VTE, vigorous prophylaxis would be the most pragmatic approach. Our patient has lost weight and has opted for anticoagulation for 1 year. Two years later, he regularly attends both the hematology and the medical outpatient clinic and remains without clinical, biochemical, or radiological evidence of myeloproliferative disorders, paroxysmal nocturnal hemoglobinuria, autoimmune, haematological, or mitotic diseases.

The main recommendations for travelers who are at high risk for PE or VTE are frequent ambulation, calf muscle exercise, or sitting in an aisle seat if feasible. The use of properly fitted, below knee graduated compression stockings providing 15–30 mm of Hg of pressure at the ankle and during travel is also recommended. The use of aspirin or anticoagulants as PE or VTE prophylaxis is not recommended [36]. Greater public education and research on risk factors for VTE or PE among travelers are needed.

In conclusion, although the extensive literature available on PE associated with air travel supports the existence of several individual risk factors for VTE, it appears that isolated ECS without any associated risk factor is a very rare event. Currently, the clinicians base their decisions on fitness to travel and need for prophylaxis on those risk factors for PE. However, our clinical case suggests that clinicians should start to make decisions on fitness to travel and encouragement of preventive measures based on the existence of combined risk factors in individualized cases even for short-haul flights. Also patients with SCT may be at risk for PE associated with travel when other risks for PE exist. These associations and considerations are novel and important in clinical practice and travel industry. Prophylactic measures for long-haul flights have been advocated for travel to minimize this risk but no risk scale has been described to guide physicians yet.
